# Drought-Induced Alterations in Carbon and Water Dynamics of Chinese Fir Plantations at the Trunk Wood Stage

**DOI:** 10.3390/plants13202937

**Published:** 2024-10-20

**Authors:** Yijun Liu, Li Zhang, Wende Yan, Yuanying Peng, Hua Sun, Xiaoyong Chen

**Affiliations:** 1 College of Life Science and Technology, Central South University of Forestry and Technology, Changsha 410004, China; christianliu8919@126.com (Y.L.); woshizl1989@126.com (L.Z.); sunhua@csuft.edu.cn (H.S.); 2 National Engineering Laboratory for Applied Forest Ecological Technology in Southern China, Changsha 410004, China; 3 College of Arts and Sciences, Lewis University, Romeoville, IL 60446, USA; pengyu@lewisu.edu; 4 College of Arts and Sciences, Governors State University, University Park, IL 60484, USA

**Keywords:** carbon fluxes, water fluxes, drought, Chinese fir plantations, climate change

## Abstract

Over the past three decades, China has implemented extensive reforestation programs, primarily utilizing Chinese fir (*Cunninghamia lanceolata* (Lamb.) Hook) in southern China, to mitigate greenhouse gas emissions and counter extreme climate events. However, the effects of drought on the carbon sequestration capacity of these forests, particularly during the trunk wood stage, remain unclear. This study, conducted in Huitong, Hunan, China, from 2008 to 2013, employed the eddy covariance method to measure carbon dioxide (CO_2_) and water fluxes in Chinese fir forests, covering a severe drought year in 2011. The purpose was to elucidate the dynamics of carbon and water fluxes during a drought year and across multi-normal year averages. The results showed that changes in soil water content (−8.00%), precipitation (−18.45%), and relative humidity (−5.10%), decreases in air temperature (−0.09 °C) and soil temperature (−0.79 °C), and increases in vapor pressure deficit (19.18%) and net radiation (8.39%) were found in the drought year compared to the normal years. These changes in environmental factors led to considerable decreases in net ecosystem exchange (−40.00%), ecosystem respiration (−13.09%), and gross ecosystem productivity (−18.52%), evapotranspiration (−12.50%), and water use efficiency (−5.83%) in the studied forests in the drought year. In this study, the occurrence of seasonal drought due to uneven precipitation distribution led to a decrease in gross ecosystem productivity (GEP) and evapotranspiration (ET). However, the impact of drought on GEP was greater than its effect on ET, resulting in a reduced water use efficiency (WUE). This study emphasized the crucial role of water availability in determining forest productivity and suggested the need for adjusting vegetation management strategies under severe drought conditions. Our results contributed to improving management practices for Chinese fir plantations in response to changing climate conditions.

## 1. Introduction

As global temperatures rise due to increased greenhouse gas emissions, the hydrological cycle intensifies, leading to significant changes in precipitation [1,2]. These alterations manifest in various forms, such as shifts in the timing, intensity, and distribution of rainfall [3]. Some regions may experience more frequent and severe droughts, while others may face increased occurrences of heavy rainfall and flooding [4]. These changes in precipitation not only affect water availability and agricultural productivity but also pose challenges to infrastructure, ecosystems, and human communities [5]. Understanding and addressing the complexities of climate change and its impact on precipitation is crucial for developing effective adaptation and mitigation strategies, particularly in the context of forests and afforestation efforts [6].

Afforestation, the process of establishing forests on lands that have not been forested for a long time or were previously used for non-forest purposes, has been recognized as a crucial strategy for mitigating climate change by sequestering atmospheric carbon dioxide (CO_2_) and enhancing ecosystem resilience to environmental stressors [7]. By capturing CO_2_ from the atmosphere through photosynthesis, trees and forests act as carbon sinks, effectively removing and storing significant amounts of carbon. This process helps to reduce the concentration of CO_2_ in the atmosphere, thereby mitigating the greenhouse effect and its associated impacts on global climate patterns [8]. Additionally, afforestation contributes to the conservation of biodiversity, soil protection, water resource management, and the provision of various ecosystem services [9]. As climate change continues to pose significant challenges to ecosystems and human societies, afforestation initiatives play an increasingly important role in adaptation and mitigation efforts, providing multiple benefits for both the environment and human well-being.

Plant growth and development are intricately linked to water availability, as water plays a fundamental role in various physiological processes essential for plant functioning [10]. Consequently, water conditions exert a significant influence on the carbon flux of ecosystems. Drought events, characterized by prolonged periods of water deficit, are closely tied to the distribution of annual precipitation patterns [11]. Insufficient precipitation input, particularly during critical growth stages, often emerges as the primary driving factor of drought stress in ecosystems [12]. The scarcity of water during drought events can disrupt photosynthesis, respiration, and other carbon cycling processes, leading to reduced productivity and altered ecosystem functioning [13].

Water use efficiency (WUE) is a measure of how efficiently a plant or an ecosystem uses water to produce biomass or perform photosynthesis [14]. In the context of forest ecosystems and carbon flux studies, WUE is often defined as the ratio of carbon gained through photosynthesis (or gross primary production, GPP) to water lost through evapotranspiration (ET) [15]. It is expressed in units such as grams of carbon per kilogram of water (gC kg^−1^ H_2_O). A higher WUE indicates that the plant or ecosystem is more efficient in using water to assimilate carbon, which is especially important in the context of drought conditions. WUE connects the carbon and water processes within forest ecosystems, not only elucidating the interrelationship between carbon and water but also highlighting the ecosystem’s sensitivity to climate change [16]. WUE is closely related to several environmental and biological factors, such as air temperature (Ta), vapor pressure deficit (VPD), and photosynthetically active radiation (PAR) [14]. Additionally, soil water content (SWC) was identified as a key determinant of WUE [17]. Understanding WUE is crucial for optimizing agricultural practices, as highlighted in a review by Medrano et al. [18]. The relationship between WUE and net radiation (Rn) in plant ecosystems is complex; Rn influences the energy available for photosynthesis and transpiration, while WUE affects the balance between carbon assimilation and water loss [19].

Chinese fir is a species of coniferous tree native to China, valued for its rapid growth, adaptability, and economic significance, making it ideal for timber production and reforestation projects in China [20]. Moreover, Chinese fir exhibits high water demand, particularly during its active growth period, highlighting its importance in regional water cycles [21]. However, the susceptibility of these forests to drought events poses a significant challenge to their carbon and water flux dynamics, particularly as climate change exacerbates the frequency and severity of droughts [22]. Understanding how Chinese fir forests respond to drought stress is crucial for developing sustainable forest management practices and climate change adaptation strategies.

Despite the recognized importance of Chinese fir in forest ecosystems, there is a notable gap in the literature regarding how these trees specifically respond to drought stress at different growth stages. The aim of this study is to evaluate the impacts of drought conditions on carbon and water dynamics in Chinese fir forests at the trunk wood stage, aimed at developing sustainable forest management practices and climate change adaptation strategies. Using the eddy covariance method, we measured ecosystem-scale exchanges of CO_2_ and water vapor in these forests in Huitong, Hunan, China, from 2008 to 2013, including a severe drought event in 2011. By analyzing carbon and water flux data alongside environmental variables, this study investigates the mechanisms underlying the response of these forests to drought and assesses their resilience under changing climatic conditions. The objectives of this study were as follows: (1) to assess the effects of drought on CO_2_ fluxes and water use efficiency (WUE), emphasizing seasonal and daily variations during the growing season; (2) to examine the influence of drought on key water fluxes, including evapotranspiration (ET), net ecosystem carbon exchange (NEE), ecosystem respiration (RE), and gross ecosystem productivity (GEP); and (3) to quantify changes in NEE, RE, and GEP between drought and non-drought periods to understand their implications for forest carbon and water dynamics.

## 2. Results

### 2.1. Daily and Seasonal Dynamics of Environmental Factors

The statistical tests performed in this study revealed significant differences between normal and drought years. Drought conditions in 2011 resulted in a slight decrease in air while soil temperature (Ts) experienced a more substantial decrease of 0.79 °C (Table 1). In contrast, the vapor pressure deficit (VPD) increased by approximately 19.2% during the drought year, indicating greater atmospheric dryness. Additionally, soil water content (SWC) decreased by 8.0% under drought conditions, which may have serious implications for overall ecosystem health. Precipitation (P) exhibited the most dramatic decrease, with an 18.5% reduction during the drought year, further contributing to water stress within the ecosystem. Relative humidity (RH) was also reduced by 5.1% under drought conditions, exacerbating the dry environment. Notably, net radiation (Rn) increased by 8.4% during the drought year, potentially influencing carbon uptake processes.

Specifically, variables related to carbon and water fluxes demonstrated significant decreases, with net ecosystem exchange (NEE), ecosystem respiration (RE), gross ecosystem productivity (GEP), and evapotranspiration (ET) all showing reductions ranging from 12.5% to 40.0% during the drought year compared to normal years (Table 1). Statistically significant differences were observed between the normal year and the drought year of 2011. For instance, NEE decreased significantly from an average of 1.45 g C m⁻^2^ day⁻^1^ in normal years to 0.87 g C m⁻^2^ day⁻^1^ during the drought year, reflecting a substantial reduction of 40.0% (*p* < 0.05). Similarly, GEP showed a marked decrease from 6.75 g C m⁻^2^ day⁻^1^ to 5.50 g C m⁻^2^ day⁻^1^, a decline of 18.5% (*p* < 0.05). Ecosystem respiration (RE) also decreased significantly, from 5.27 g C m⁻^2^ day⁻^1^ to 4.58 g C m⁻^2^ day⁻^1^, which is a 13.1% reduction (*p* < 0.05). Furthermore, ET was reduced from 2.32 mm to 2.03 mm, signifying a 12.5% decrease in water vapor during the drought period (*p* < 0.05). Water use efficiency (WUE), measured as grams of carbon per kilogram of water, decreased by 5.83% from 3.26 g C kg⁻^1^ H_2_O to 3.07 g C kg⁻^1^ H_2_O, with this difference also statistically significant (*p* < 0.05).

**Figure 1:**
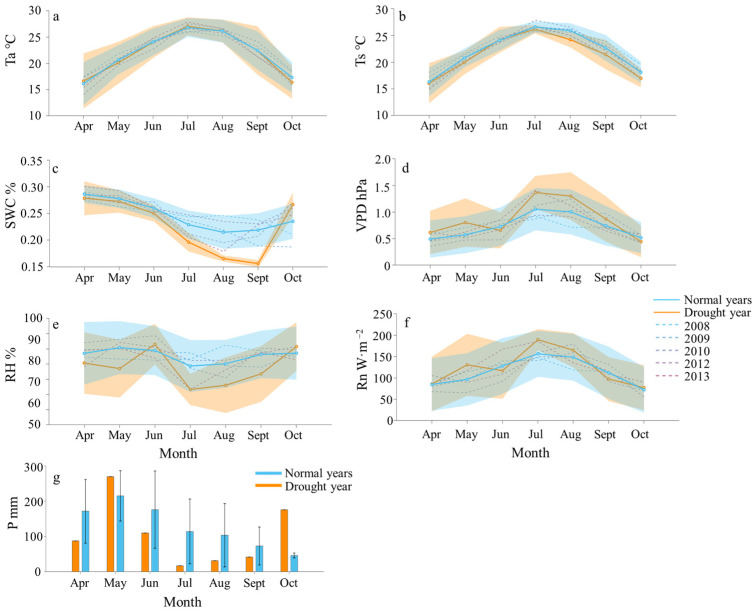
Dynamics of environmental factors during the growing season in a drought year in the drought year (2011, orange) compared to the normal years (average of 2008, 2009, 2010, 2012, 2013; blue): (**a**) air temperature (Ta), (**b**) soil temperature (Ts), (**c**) soil moisture content (SWC), (**d**) saturated vapor pressure deficit (VPD), (**e**) relative humidity (RH), (**f**) net radiation (Rn), and (**g**) precipitation (P). The solid lines with dots represent the monthly average daily values, and the shaded regions indicate the standard deviation for the drought year (orange) and normal years (blue). Dashed lines represent the monthly average daily values for different years within the normal year range. Bars represent the cumulative monthly precipitation, with error bars indicating one standard error.

The monthly average daily values of the main environmental factors for the growing season in the drought year of 2011 and the normal years are shown in Figure 1. Both Ta and Ts exhibited an initial rise followed by a decline, reaching their peaks in July across all the study years. In the drought year, the highest Ta and Ts were 27.07 and 25.98 °C, respectively, slightly surpassing the normal year’s values of 26.83 and 25.62 °C. The lowest Ta and Ts in the drought year were recorded in October (16.64 °C) and April (15.60 °C), respectively. In contrast, the lowest Ta and Ts in the normal years were both observed in April, at 16.12 and 15.24 °C, respectively (Figure 1a,b). In contrast to Ta and Ts, SWC demonstrated a pattern of initial decline followed by an increase during the drought year of 2011, exhibiting lower values compared to the normal years. The lowest value in the drought year of 2011 occurred in September, at 15.57%, while the highest was recorded in April, at 27.82%. Conversely, the lowest SWC value in the normal years was observed in August, at 21.44%, and the highest in April, at 28.60% (Figure 1c). The trend in VPD mirrors that of temperature (Ta and Ts), with peak values in both the normal years and the drought year occurring in July, at 1.05 hPa and 1.37 hPa, respectively. The lowest VPD value in the normal years was noted in April, at 0.49 hPa, whereas, in the drought year, it occurred in October, at 0.44 hPa (Figure 1d). RH exhibited similarities across different years, yet it fluctuated more prominently in the drought year, consistently remaining lower than the normal years in all months except June. The highest RH during the drought year of 2011 was noted in June, at 81.53%, while the lowest value occurred in July, at 66.67%. The highest RH in the normal years was recorded in June, at 80.41%, with the lowest in July, at 74.37% (Figure 1e). Rn during the growing season in the drought year surpassed that of the normal years. The maximum Rn value of the growing season during the drought year and the normal years both occurred in July, at 189.56 and 156.52 W·m^−2^, respectively, while the minimum values were observed in October, at 77.11 and 72.08 W·m^−2^, respectively (Figure 1f). In the drought year of 2011, precipitation (P) markedly decreased compared to the normal years, with the highest monthly cumulative quantity value in the normal years recorded in May, at 215.36 mm, and the lowest in October, at 46.31 mm. Conversely, the highest monthly cumulative quantity value of P in the drought year occurred in May, at 269.83 mm, while the lowest occurred in July, at merely 16.90 mm. A prolonged dry spell of 24 consecutive days without P was observed in July (Figure 1g).

### 2.2. Seasonal Dynamics of Carbon and Water Fluxes

**Figure 2:**
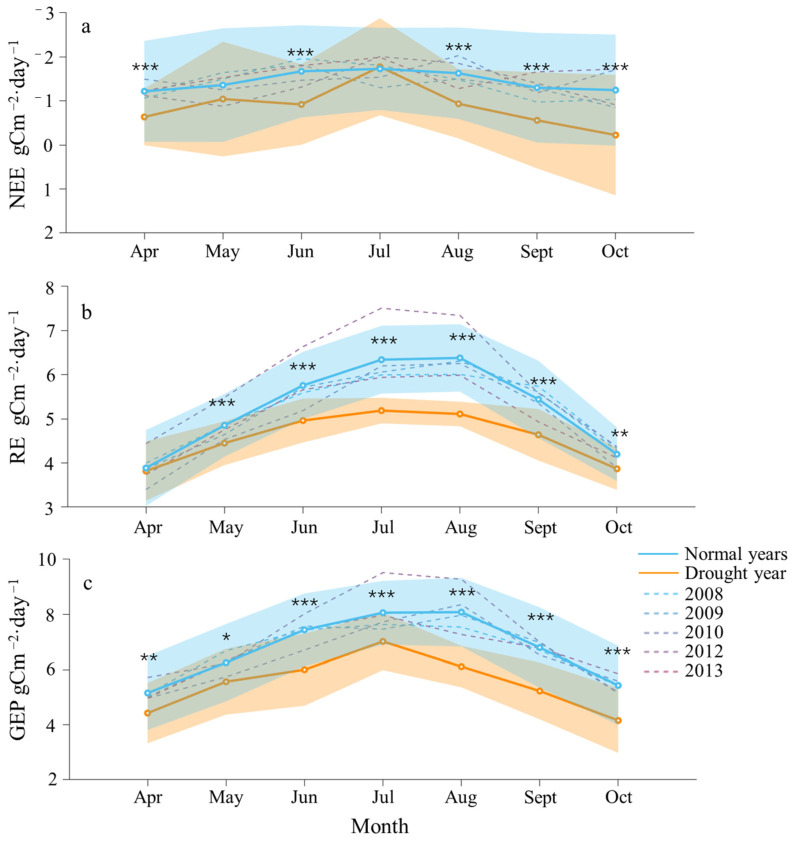
Dynamics of carbon flux components during the growing season in the drought year (2011, orange) compared to normal years (average of 2008, 2009, 2010, 2012, and 2013; blue): (**a**) net ecosystem exchange (NEE), (**b**) ecosystem respiration (RE) and (**c**) gross ecosystem productivity (GEP). The solid lines with dots represent the monthly average daily values, while the translucent fill indicates the standard deviation for the drought year (orange) and normal years (blue). The dashed line represents the monthly average daily values for individual normal years. Asterisks denote the significance of differences between the datasets, with * indicating *p* < 0.05, ** indicating *p* < 0.01, and *** indicating *p* < 0.001.

The average daily values of NEE during the growing season exhibited relative stability in the normal years, but they showed greater fluctuations in the drought year of 2011 (Figure 2). Additionally, NEE was consistently lower in the drought year than in the normal years. The lowest mean daily values of NEE across different years all occurred in July, registering at −1.78 gCm^−2^·day^−1^ in the drought year and −1.73 gCm^−2^·day^−1^ in the normal years. Conversely, the highest mean daily value of NEE was −1.22 gCm^−2^·day^−1^ observed in April in the normal years, and it was −0.23 gCm^−2^·day^−1^ observed in October in the drought year (Figure 2a).

The average daily values of RE and GEP during the growing season in different years exhibited a similar pattern of initially increasing and then decreasing, with values generally lower in the drought year compared to the normal years. The maximum values of both in the normal years were recorded in August, at 8.08 and 6.38 gCm^−2^·day^−1^, respectively. Conversely, the lowest values occurred in April, at 5.15 and 3.89 gCm^−2^·day^−1^, respectively. In the drought year of 2011, the maximum values for both RE and GEP were observed in July, at 7.02 and 5.19 gCm^−2^·day^−1^, respectively, while the lowest values occurred in April (4.15 gCm^−2^·day^−1^) and October (3.82 gCm^−2^·day^−1^) (Figure 2b,c).

**Figure 3:**
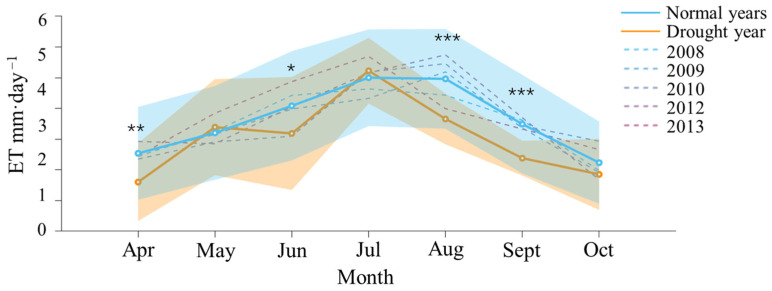
Dynamics of evapotranspiration (ET) during the growing season in the drought year (2011, orange) compared to normal years (average of 2008, 2009, 2010, 2012, and 2013; blue). The solid lines with dots represent the monthly average daily values, and the translucent fill indicates the standard deviation for the drought year (orange) and normal years (blue). The dashed line represents the monthly average daily values for individual normal years. Asterisks denote the significant levels of differences between the two datasets, with * indicating *p* < 0.05, ** indicating *p* < 0.01, and *** indicating *p* < 0.001.

**Figure 4:**
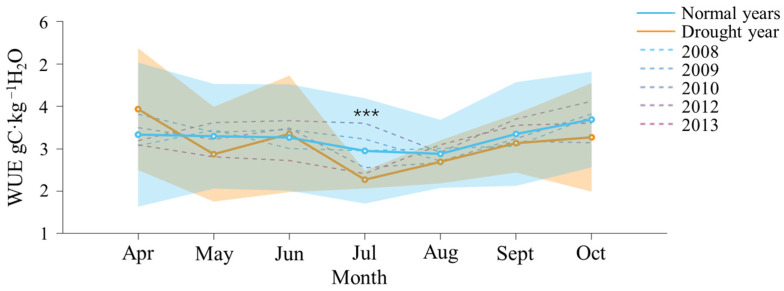
Dynamics of water use efficiency (WUE) during the growing season in the drought year (2011, orange) compared to normal years (average of 2008, 2009, 2010, 2012, and 2013; blue). The solid lines with dots represent the monthly average daily values, and the translucent fill indicates the standard deviation for the drought year (orange) and normal years (blue). The dashed line represents the monthly average daily values for individual normal years. Asterisks denote the significant levels of differences between the two datasets, with *** indicating *p* < 0.001.

Except for May and July, ET in the drought year was lower than that in the normal years and exhibited greater fluctuations (Figure 3). The overall trend across different years was to initially increase and then decrease. The highest values of ET in both the drought year and the normal years occurred in July, at 3.11 and 3.00 mm·d^−1^, respectively. The lowest value in the drought year of 2011 occurred in April, at 1.30 mm·d^−1^, while in the normal year, it occurred in October, at 1.62 mm·d^−1^. WUE in the drought year was lower than that in the normal year and exhibited greater fluctuations, except for April and June (Figure 4). In contrast, WUE in the normal years was generally more stable. The maximum and minimum values of WUE in the normal year occurred in October (3.7 gC·kg^−1^H_2_O) and August (2.9 gC·kg^−1^H_2_O). The maximum and minimum values of WUE in the drought year occurred in April (3.9 gC·kg^−1^H_2_O) and July (2.3 gC·kg^−1^H_2_O).

### 2.3. Differences in Carbon–Water Flux and Environmental Factors

**Figure 5:**
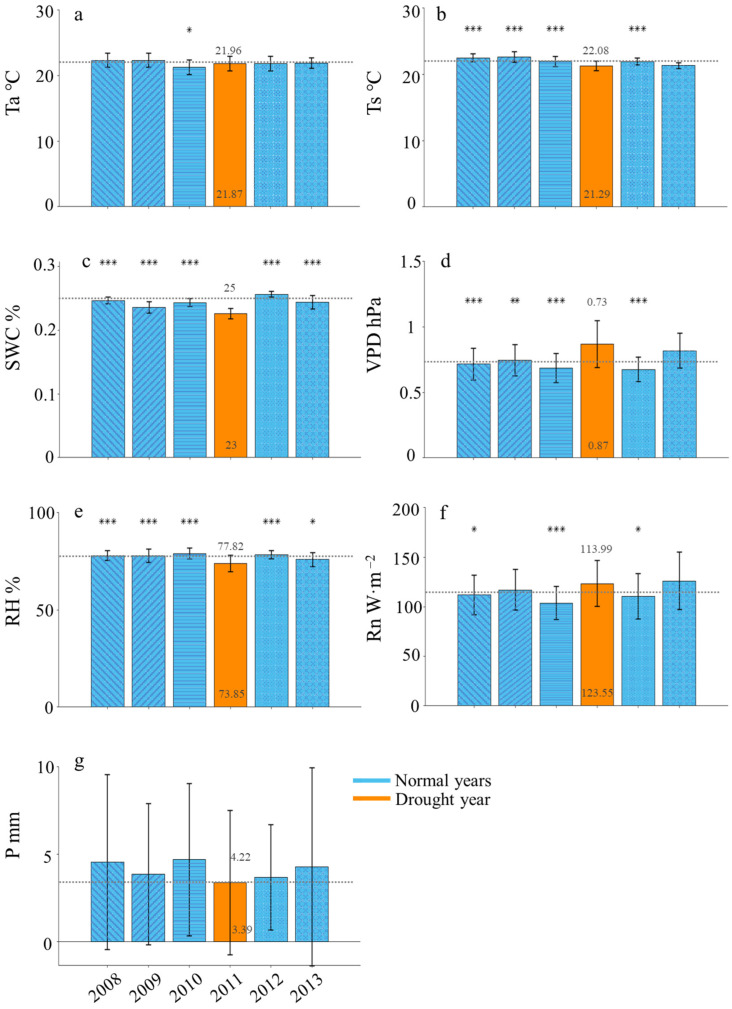
Comparison of daily average values of environmental factors during the growing seasons between the drought year (2011) and the normal years (2008, 2009, 2010, 2012, 2013; blue): (**a**) air temperature (Ta), (**b**) soil temperature (Ts), (**c**) soil moisture content (SWC), (**d**) saturated vapor pressure deficit (VPD), (**e**) relative humidity (RH), (**f**) net radiation (Rn), and (**g**) precipitation (P). The black short lines are error bars. The dashed line across all bars represents the average of normal years. Numbers above the dashed line correspond to the average of normal years, while numbers below represent values from the drought year. Asterisks denote the significance of differences between the drought year and the various normal years, with * indicating *p* < 0.05, ** indicating *p* < 0.01, and *** indicating *p* < 0.001.

There was no significant difference between the normal years and drought year in Ta (Figure 5a). The Ts during drought year was significantly lower than that of normal years; however, there was no significant difference in Ts between the drought year and 2013, (Figure 5b). All other water factors showed significant differences compared to the normal years. Specifically, the soil water content (SWC) and vapor pressure deficit (VPD) were higher in the drought year, while relative humidity (RH) was lower than in the normal years. (Figure 5c–e). The VPD during drought year was significantly higher than that of normal years; however, there was no significant difference in the VPD between the drought year and 2013. The Rn during drought year was generally higher than that of normal years but slightly lower than in 2013. Additionally, the Rn in drought years was significantly greater than in the years 2008, 2010, and 2012. (Figure 5f). P showed no significant difference between the drought year and the normal years (Figure 5g).

**Figure 6:**
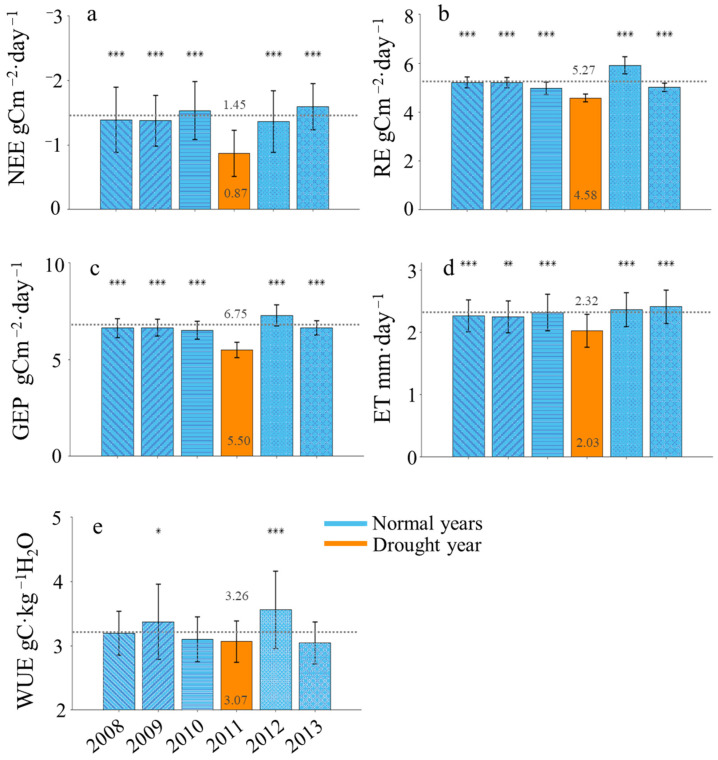
Comparison of daily average values of carbon and water fluxes during the growing seasons between the drought year (2011, orange) and the normal years (2008, 2009, 2010, 2012, 2013; blue): (**a**) net ecosystem exchange (NEE), (**b**) ecosystem respiration (RE), (**c**) gross ecosystem productivity (GEP), (**d**) evapotranspiration (ET), and (**e**) water use efficiency (WUE). The black short lines are error bars. The dashed line across all bars represents the average of normal years. Numbers above the dashed line correspond to the average of normal years, while numbers below represent values from the drought year. Asterisks denote the significance of differences between the drought year and the various normal years, with * indicating *p* < 0.05, ** indicating *p* < 0.01, and *** indicating *p* < 0.001.

There were significant differences in the components of the growing season carbon flux between different years, and all components of the carbon flux in the drought year were significantly lower than in the normal years (Figure 6). The decrease in NEE in the drought year is particularly pronounced. In the drought year, NEE was only 60% of the average for normal years. NEE gradually increased thereafter, reaching 94.5% of the normal years’ average in 2012 and increasing by 10% compared to the normal average in 2013. RE during the drought year was 86.9% of the normal years’ average, with a noticeable rise in 2012, increasing by 12.3% compared to the normal years’ average. In 2013, RE decreased to 95.3% of the normal years’ average, but it was still slightly higher than in 2010 (an increase of 0.8%). GEP in the drought year was 81.4% of the normal years’ average. In 2012, GEP significantly increased, rising by 8% compared to the normal years’ average, while in 2013, it fell back to 98.5% of the normal years’ average, remaining slightly above 2010 (an increase of 2.0%). ET during the drought year was 87.5% of the normal years’ average, followed by a gradual increase. In 2012, ET increased by 1.72% compared to the average of normal years, and in 2013, it increased by 3.9%. WUE during drought years was lower than in normal years, with significant differences observed only compared to the years 2009 and 2012, at 94.17% of the normal years’ average. There was a significant increase in 2012, rising by 9.2%. In 2013, WUE decreased to 93.6%, compared to 98.4% in 2010. Additionally, ET and WUE in the drought year were also significantly lower than in the normal years (Figure 6).

From daily averages compared to normal years in the drought year, the average Ta decreased by 0.09 °C, Ts decreased by 0.79 °C, VPD increased by 19.18%, and SWC decreased by 8.00%. RH decreased by 5.10%, and Rn increased by 8.39%. The monthly cumulative precipitation (P) in the drought year decreased by 18.45% compared to the normal years. Additionally, average daily carbon and water fluxes in the drought year decreased to some extent relative to normal years. NEE declined by 40.00%, RE decreased by 13.09%, GEP decreased by 18.52%, ET decreased by 12.50%, and WUE decreased by 5.83%.

During the growing season, the NEE accumulation in normal years and the drought year was −291.40 and −195.14 gCm^−2^·a^−1^, respectively, with a decrease of 33.03% in drought year. The RE accumulation was 1132.80 and 1033.43 gCm^−2^·a^−1^, respectively, with a decrease of 8.77% in the drought year. The GEP accumulation was 1429.82 g Cm^−2^·a^−1^ during the normal years’ average and 1228.56 gCm^−2^·a^−1^ during the drought year, reflecting a decrease of 14.08% in the drought year compared to the normal years’ average. The ET accumulation in normal years and drought year were 572.82 and 586.01 mm, respectively, with an increase of 2.3% in the drought year. The WUE in normal years and the drought year during the growing season was 3.26 and 3.07 gC·kg^−1^H_2_O, respectively, with a decrease of 5.83% in the drought year (Figure 6).

## 3. Discussion

### 3.1. Carbon-Water Fluxes

Our research indicated that drought conditions significantly impacted environmental variables and ecosystem processes. The slight decrease in air temperature (Ta) by 0.09 °C and a more substantial decrease in soil temperature (Ts) by 0.79 °C suggest that soil thermal dynamics are sensitive to drought-induced changes, likely due to reduced soil moisture and altered heat fluxes. This observation aligns with previous findings that noted a decrease in Ta during the drought year compared to normal ones [23]. The increase in VPD by 19.18% during the drought year highlights the heightened atmospheric dryness, exacerbating conditions for plant water uptake and transpiration [24]. This is compounded by an 8.00% decrease in SWC [25,26], underscoring the severity of water stress within the ecosystem. Additionally, the 5.10% decline in RH [27] emphasizes the aridification associated with prolonged drought events. Interestingly, despite these adverse conditions, Rn increased by 8.39% during the drought year [28], likely due to reduced cloud cover and altered atmospheric conditions. The most striking change was in P, which saw a reduction of 18.45% during the drought year [29,30], critically affecting water availability for sustaining ecological processes.

Moreover, ecosystem functioning variables, including NEE, RE, GEP, and ET, exhibited substantial decreases ranging from 12.50% to 40.00% during the drought year [31,32]. This decline underscores the profound impact of water scarcity on ecosystem processes and the vulnerability of ecosystems to prolonged drought events. The 5.83% decrease in water use efficiency (WUE) during drought conditions highlights the challenge of optimizing resource utilization under water stress. This finding is consistent with Zhang et al. [33], who emphasized species-specific adaptations in subtropical forests during drought, and Mi et al. [34], who reported significant reductions in WUE in coniferous forests across China, discussing the sensitivity of WUE components (GPP and ET) to the response of comprehensive external environmental conditions. These studies illustrate the variability of WUE responses across forest ecosystems, enhancing the context for understanding the impacts of drought on forest dynamics [35].The comparative analysis of environmental factors between the drought year and the normal years’ average reveals significant variations in key parameters impacting the ecosystem dynamics. Ta and Ts exhibited a typical seasonal pattern, peaking in July, with the drought year of 2011 experiencing slightly higher peak temperatures than normal years, consistent with previous studies indicating increased temperatures during drought periods [23]. In contrast, soil water content (SWC) is lower in the drought year, highlighting the impact of drought conditions on soil moisture levels, which can influence plant water uptake and ecosystem functioning [36]. Vapor pressure deficit (VPD) closely corresponds with temperature trends, peaking during the summer months. The pronounced decrease in precipitation (P) during the drought year underscores the severity of drought conditions, potentially leading to water stress and reduced ecosystem productivity [13]. Furthermore, fluctuations in relative humidity (RH), particularly the notable decrease in the drought year, emphasize the aridity associated with drought conditions. Radiation (Rn) during the growing season in the drought year surpasses that of normal years, indicating higher energy input, which may exacerbate water loss through evapotranspiration and contribute to water stress in the ecosystem [37].

The seasonal dynamics of carbon and water fluxes provide insights into forest ecosystems’ responses to climatic variability, particularly during the drought year. This observation aligns with other studies indicating that droughts significantly reduce forest carbon uptake and impact water fluxes, altering ecosystem productivity and resilience to climatic stress [38,39]. The pronounced fluctuations in net ecosystem exchange (NEE) during the growing season, with greater variability and consistently lower values in the drought year, highlight the vulnerability of carbon dynamics to water stress. These results echo previous research showing the adverse effects of drought on carbon sequestration and ecosystem productivity [40]. The highest NEE values recorded in July across years indicate a critical period of reduced carbon uptake, likely due to heightened water limitations and increased respiratory losses, consistent with findings that high summer temperatures and drought conditions lead to increased respiration and decreased carbon sequestration [41,42]. In contrast, lower NEE values in April during the normal years suggest enhanced carbon assimilation under favorable conditions, where early-season growth coincides with optimal soil moisture and temperature levels [38].

The monthly average daily values of RE and GEP display a consistent seasonal pattern across years, initially increasing during the early growing season and then declining. Notably, values for both RE and GEP were lower during the drought year compared to a normal year. In a typical year, peak values were recorded in August at 8.08 gCm^2^·day⁻^1^ for RE and 6.38 gCm^2^·day⁻^1^ for GEP, while the lowest values were in April at 5.15 gCm^2^·day⁻^1^ and 3.89 gCm^2^·day⁻^1^, respectively. In contrast, during the drought year, maximum RE and GEP occurred earlier in July, reaching 7.02 gCm^2^·day⁻^1^ and 5.19 gCm^2^·day⁻^1^, respectively, with the lowest values observed in April and October at 4.15 gCm^2^·day⁻^1^ for RE and 3.82 gCm^2^·day⁻^1^ for GEP. The earlier peak in GEP during the drought year indicates that drought conditions significantly impacted carbon dynamics in Chinese fir plantations, suggesting that climatic anomalies play a crucial role in altering seasonal carbon flux cycles within forest ecosystems [43].

The monthly average daily values of ET during the growing season show significant interannual variability and seasonal trends (Figure 3). ET was generally lower in the drought year compared to normal years, except for May and July, indicating a more unstable water balance likely influenced by climatic factors and precipitation variations. Both drought and normal years exhibited a similar seasonal pattern, with ET increasing early in the growing season and peaking in July. The peak ET recorded in July was 3.11 mm·d⁻^1^ for the drought year and 3.00 mm·d⁻^1^ for normal years, highlighting the mid-summer maximum typical of temperate forest ecosystems [44]. The lowest ET in the drought year occurred in April (1.30 mm·d⁻^1^), while in normal years, the minimum was in October (1.62 mm·d⁻^1^). These variations suggest that the growing season during the drought year experienced atypical conditions, potentially leading to reduced water availability and altered plant physiological responses [45]. Drought-induced water stress can result in hydraulic failure, impairing plants’ ability to transport water and affecting photosynthesis and carbon assimilation [46]. Prolonged drought may also cause carbon starvation, as plants struggle to produce sufficient carbohydrates for vital functions [47].

### 3.2. Water Use Efficiency

Figure 5 presents the monthly average daily values of WUE during the growing season, revealing significant differences between the drought year and normal years. The WUE in the drought year was consistently lower than in normal years, with greater fluctuations, indicating less favorable conditions for maintaining a stable WUE. In normal years, WUE stability is highlighted by maximum and minimum values recorded in October (3.7 gC·kg⁻^1^ H_2_O) and August (2.9 gC·kg⁻^1^ H_2_O), reflecting consistent water utilization by crops throughout the growing season. This consistency is crucial for optimizing agricultural water use, ensuring crops receive adequate water while minimizing wastage, especially under varying environmental conditions [48]. A stable WUE allows crops to maximize carbon uptake relative to water use, vital for maintaining growth during low water availability, particularly in regions with seasonal fluctuations [47]. Additionally, a stable WUE helps reduce water losses due to inefficient usage, balancing water availability and crop needs during critical growth phases [49]. In contrast, the drought year exhibited higher variability in WUE, with maximum and minimum values in April (3.9 gC·kg⁻^1^ H_2_O) and July (2.3 gC·kg⁻^1^ H_2_O), suggesting erratic water use efficiency. This variability may stem from factors such as climatic conditions, soil moisture differences, or changes in crop management practices. The peak in April may indicate optimal conditions for water use, while the dip in July likely corresponds to stress from high temperatures or water scarcity. Climatic variability and soil moisture levels significantly impact water use efficiency in crops [50].

### 3.3. The Water Use Efficiency and the Environmental Factors

The differences in WUE and environmental factors between the drought year and normal years illustrate the complex interactions between climatic conditions and plant physiological responses. The lower and more fluctuating WUE in the drought year suggests that plants encountered challenging growing conditions, likely due to variations in soil water content (SWC), relative humidity (RH), and vapor pressure deficit (VPD). Lower Ts and higher Rn, along with lower SWC and RH, increased water stress, reducing plants’ efficiency in utilizing water for biomass production [51]. These observations align with research showing that increased temperatures and reduced soil moisture significantly impact WUE by altering transpiration rates and water uptake efficiency [24,52]. Furthermore, a higher VPD in the drought year intensified atmospheric water demand, compounding plant stress and leading to a reduced WUE [53]. Understanding these dynamics is crucial for developing adaptive management strategies to enhance crop resilience under varying climatic conditions and ensure sustainable agricultural productivity [51].

In subtropical primitive forests and alpine wetland ecosystems, water conditions primarily drive WUE [54]. The significant differences in carbon flux, evapotranspiration (ET), and WUE across years, as shown in Figure 6, highlight the impact of climatic variations on ecosystem functioning. The considerable decrease in all carbon flux components during the drought year reflects reduced ecosystem productivity, particularly evident in the decline of NEE, underscoring the sensitivity of carbon sequestration processes to environmental factors like temperature and soil moisture [38,55]. Water stress limits carbon dioxide assimilation, mainly due to stomatal closure aimed at preventing xylem blockage [56]. The notably lower ET and WUE during the drought year indicate the adverse effects of altered climatic conditions on water balance and plant physiology. Reduced ET suggests lower water transpiration rates from vegetation, potentially impacting cooling effects and regional climate patterns [57]. The decrease in WUE highlights the challenges plants face in optimizing carbon assimilation relative to water loss under unfavorable conditions [39]. These findings echo previous studies on ecosystem vulnerability to climate variability and emphasize the need for effective management and adaptation strategies to mitigate climate change impacts on ecosystem functioning and services [40,58]. The observed changes in environmental parameters during the drought year significantly affect ecosystem functioning and water balance, with decreased temperature and SWC alongside increased VPD, indicating heightened water stress [59,60]. This change can lead to reduced plant water availability and increased transpiration loss, affecting productivity and carbon dynamics [61]. The substantial decrease in P and RH exacerbates water scarcity, impacting plant growth and WUE [42]. The increase in Rn suggests altered energy balance dynamics, influencing RE, GEP, and NEE of carbon [62]. These results underline ecosystems’ vulnerability to drought and highlight the necessity for adaptive management strategies to mitigate climate change impacts on functioning [20].

This study’s findings have significant global implications for afforestation projects aimed at mitigating climate change. Understanding how forest ecosystems respond to climatic variability, particularly drought, is crucial as nations ramp up their afforestation efforts. The observed reductions in carbon fixation and WUE during drought conditions highlight the vulnerability of these initiatives to water stress, emphasizing the need for drought-resistant species and adaptive management strategies [34,63]. Irregular precipitation patterns can exacerbate water stress, leading to declines in GEP and reduced carbon sink capacity, which may hinder the objectives of afforestation [64,65]. By integrating insights from this study, stakeholders can enhance the resilience of newly established forests, ensuring effective carbon sequestration while promoting biodiversity conservation [66]. Furthermore, understanding and managing the factors affecting WUE, especially in the context of changing climates, is vital for sustainable agricultural practices in water-scarce regions. Future research should focus on identifying the drivers of WUE variability and developing strategies to maintain high productivity under adverse conditions. Ultimately, comprehending the interplay between climatic conditions and forest productivity is essential for formulating sustainable afforestation policies that contribute to both climate change mitigation and ecosystem health.

## 4. Materials and Methods

### 4.1. Study Area

**Figure 7:**
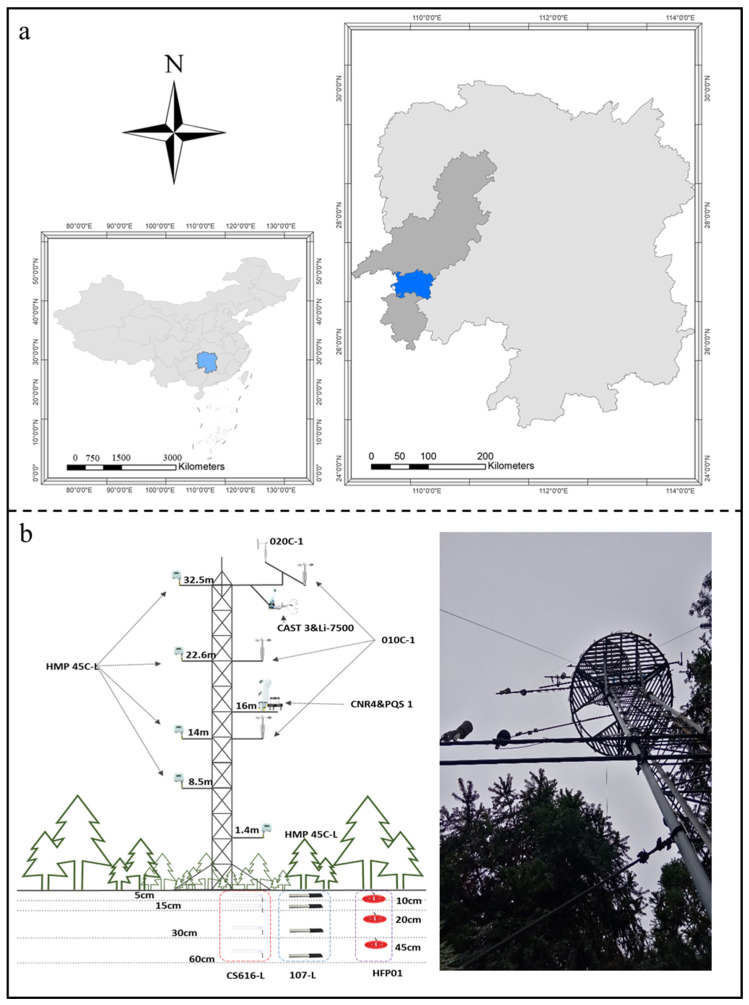
The geographic location of the National Field Scientific Observation and Research Station of Chinese Fir Forest Ecosystem in Huitong, Hunan Province of China: (**a**) the geography of the study site and (**b**) the schematic diagram of flux and meteorological gradient observation system.

This study was conducted in the National Field Scientific Observation and Research Station of Chinese Fir Forest Ecosystem in Huitong, Hunan Province, China (27.725°–28.045° N, 09.595° E to 110.015° E) (Figure 7a). The climate, characterized by a subtropical monsoon pattern, ensures ample precipitation during the warm, humid summers and a drier, cooler winter season. The rainy season typically spans from April to September, with peak rainfall occurring between May and July, contributing to over 60% of the annual precipitation. This unique climatic regime, coupled with the fertile red soils that typify the region, provides an optimal environment for the growth and development of Chinese fir, a dominant species in the area’s forestry landscape.

The Chinese fir plantations were planted in 1996, and, at the time of this study, their ages ranged from 12 to 18 years, corresponding to the trunk wood stage. The dense canopy cover of these plantations creates a microclimate that enhances moisture retention and supports biological diversity, while also functioning as a vital carbon sink and watershed protection zone. In this context, studying water use efficiency in Chinese fir plantations in Huitong provides valuable insights into the complex interactions between environmental factors, forest management practices, and ecosystem functioning within subtropical forest ecosystems.

### 4.2. Data Acquisition and Preprocessing

Seven meteorological factors including Ta, Ts, SWC, P, VPD, RH, and Rn were selected to analyze the carbon and water fluxes of Chinese fir plantations in a drought year (2011) and normal years (2008, 2009, 2010, 2012, 2013), as well as their main influencing factors.

The flux data were measured by the eddy covariance system installed on the flux tower in the catchment area. This system consists of a three-dimensional ultrasonic anemometer (CAST3, Campbell & Company, Logan, UT, USA) installed on the boom of the flux tower at 32.5 m and an open-path CO_2_/H_2_O infrared analyzer (Li-7500, Licor, Lincoln, NE, USA) (Figure 7b). The data collection was managed by a data logger (CR1000, Campbell & Company, USA), with a sampling frequency set to 10 Hz. The eddy covariance system automatically calculated the average flux data every 0.5 h, and the PC card stored both the raw data and calculation results. The sampling frequency for these sensors was set to 0.5 Hz, and the eddy covariance system automatically calculated the average flux data every 5 min.

The meteorological gradient observation system consisted of several sensors installed at various heights on the flux tower. This setup included five air temperature and humidity sensors (HMP45C-L, Vaisala, Helsinki, Finland) positioned at 1.4 m, 8.5 m, 14 m, 22.6 m, and 32.5 m to measure vertical gradients in temperature and humidity within and above the forest canopy. These measurements are crucial for understanding how these variables change with altitude, which impacts microclimate and ecosystem processes. Three wind speed sensors (010C-1, MetOne, Troy, MI, USA) were located at 14 m, 22.6 m, and 32.5 m to monitor wind speeds at different levels, providing insights into wind patterns and their influence on turbulence and dispersion within the canopy. A wind direction sensor (020C-1, MetOne, USA) at 32.5 m records the direction of wind flow at the upper canopy level, essential for understanding wind’s impact on canopy interactions and meteorological conditions. Additionally, a net radiometer (CNR4, Kipp & Zonen, Delft, The Netherlands) and a photosynthetically active radiation sensor (PQS1, Kipp & Zonen, Delft, The Netherlands) were situated at 16 m to measure radiation levels at an intermediate height. This height is critical for evaluating light availability and energy balance within the canopy, which affects photosynthesis and other biological processes. By setting the sensors at these various heights, the system provided a comprehensive profile of atmospheric conditions, enabling the detailed analysis of vertical gradients and their effects on forest microclimate and ecosystem dynamics.

### 4.3. Calculation of Carbon Flux

Since photosynthesis stops at night, the NEE value observed by the eddy covariance system can be considered as the nighttime ecosystem respiration value. By constructing a model and fitting the nighttime ecosystem respiration values with temperature variations, two parameters, the base respiration rate (*R_ref_*), and the temperature sensitivity of respiration (*E*_0_) can be estimated. By extrapolating these two parameters to the daytime, the daytime ecosystem respiration can be estimated. The temperature sensitivity model of *RE* can be represented as follows:

(1) $$RE=R_{\mathrm{ref}}\exp(E_{0}(\frac{1}{T_{\mathrm{ref}}-T_{0}}-\;\frac{1}{T_{a}-T_{0}}))\;$$

where *R_ref_* is the base respiration rate at the reference temperature (*T_ref_*), with units of μmol·C m^−2^·s^−1^; the reference temperature *T_ref_* is generally set to 15 °C; *E*_0_ (°C) can be considered the temperature sensitivity of ecosystem respiration; *T_a_* (°C) is the air temperature; and *T*_0_ is a constant, usually set to −46.02 °C. The ecosystem respiration during both day and night is estimated by the parameters *R_ref_* and *E*_0_, which are fitted from the nighttime data.

The daytime GPP can be calculated by the difference between RE and NEE. This study follows the FLUXNET methodology, utilizing a short-time sliding window (14 days) for parameter fitting to ensure uniform parameterization [67].

### 4.4. Calculation of ET

*ET* was calculated using the energy flux observed by the eddy covariance system [68]:

(2) $$\mathrm{ET}=\frac{0.43\mathrm{LE}}{\left(597\;-\;0.564T\right)}$$

where the unit of latent heat flux (*LE*) is (W·m^−2^), (597 − 0.564*T*) represents the heat of vaporization of water (cal·g^−1^), 0.43 is the unit conversion coefficient, *T* denotes the air temperature at the canopy height (°C), and the sum of each 0.5 h value in a day yields ET, with the output unit being mm·d^−1^.

### 4.5. Calculation of WUE

The *WUE* is determined by using Equation (3) as follows:

(3) $$\mathrm{WUE}=\frac{\mathrm{GEP}}{\mathrm{ET}}$$

In Equation (3), utilize the *GEP* and *ET* of the corresponding time scale to calculate the *WUE* across various time scales.

### 4.6. Average Values

The monthly average daily value was calculated by summing the daily averages for the month and dividing by the number of days in that month.

The daily average value for the growing season was calculated by summing the daily mean values for the entire growing season and dividing by the total number of days in the growing season.

### 4.7. Statistical Analysis

In this study, one-way analysis of variance (One-way ANOVA) is employed to analyze the differences in carbon–water flux, water use efficiency, and environmental factors during the growing season of different years. Post hoc tests such as Tukey’s HSD may be conducted to further investigate significant differences between specific groups. Additionally, correlation analysis or regression analysis may be employed to explore relationships between carbon–water fluxes, water use efficiency, and environmental variables.

## 5. Conclusions

This study highlights the significant impacts of drought on carbon and water fluxes in a Chinese fir forest, as assessed using eddy covariance (EC) technology. The results demonstrate that drought conditions led to substantial reductions in carbon fixation, evapotranspiration (ET), and water use efficiency (WUE) compared to normal years. Despite comparable total precipitation levels, the irregular distribution of rainfall resulted in seasonal drought, exacerbating water stress and causing a more pronounced decline in gross ecosystem productivity (GEP) than in ecosystem respiration (RE). The decreased soil water content (SWC), lower relative humidity (RH), and increased vapor pressure deficit (VPD) were key factors contributing to reduced productivity and WUE during the drought year. Furthermore, the observed fluctuations in WUE underscore the complex responses of vegetation to prolonged water stress, revealing dynamic adaptation mechanisms employed by the forest. These findings underscore the necessity of developing effective management strategies to mitigate the adverse effects of climate change on forest ecosystems. By enhancing our understanding of the carbon–water coupling dynamics and resilience strategies in Chinese fir forests, this study contributes valuable insights to the scientific community and informs future research on ecosystem responses to drought stress. Specific management strategies derived from these findings, such as thinning practices or irrigation techniques to enhance drought resilience in Chinese fir plantations, are essential for fostering sustainable forest management under changing climatic conditions.

## Figures and Tables

**Table 1:** Average daily values of environment factors and carbon and water fluxes in the examined Chinese fir plantations during growing seasons between the drought year and the normal years.

Items/Unit	The Drought Year	The Normal Years	Relative Change
Ta/°C	21.87 a	21.96 a	−0.09 °C
Ts/°C	21.29 a	22.08 b	−0.79 °C
VPD/hPa	0.87 a	0.73 b	19.18%
SWC/%	0.23 a	0.25 b	−8.00%
P/mm	3.39 a	4.22 a	−18.45%
RH/%	73.85 a	77.82 b	−5.10%
Rn W·m^−2^	123.55 a	113.99 b	8.39%
NEE/gCm^−2^·day^−1^	0.87 a	1.45 b	−40.00%
RE/gCm^−2^·day^−1^	4.58 a	5.27 b	−13.09%
GEP/gCm^−2^·day^−1^	5.50 a	6.75 b	−18.52%
ET/mm	2.03 a	2.32 b	−12.50%
WUE/gC·kg^−1^H_2_O	3.07 a	3.26 b	−5.83%

Note: Ta: air temperature; Ts: soil temperature; VPD: vapor pressure deficit; SWC: soil water content; P: precipitation; RH: relative humidity; Rn: net radiation; NEE: net ecosystem exchange; RE: ecosystem respiration; GEP: gross ecosystem productivity; ET: evapotranspiration; WUE: water use efficiency. The same lowercase letter (a) in columns indicates no significant difference between the drought year and the average of normal years (*p* > 0.05), while different lowercase letters (a and b) in columns indicate significant differences between the drought year and the average of normal years (*p* < 0.05).

## Data Availability

The data presented in this study are available upon request from the corresponding author.
